# EMSOS Congress and Symposium of the Nurse Group Program

**DOI:** 10.1080/135771402753667637

**Published:** 2002-05


					Sarcoma (2002) S1, S3-5
ISSN 1357-714X print; 1369-1643 online/02/S1/S3-5 (c) 2002 Taylor & Francis Ltd
EMSOS Congress and Symposium of the Nurse Group Program
Wednesday, 22 May 2002
14:30-17:00 Registration of EMSOS and Nurse Group participants at the Faculty Club, Rapenburg 6 (Leiden city centre).
16:30-17:45 Joint welcome reception by the Mayor of Leiden and the Board of Leiden University at the Town Hall.
Thursday, 23 May 2002
08:00 Registration desk open and mounting of posters
EMSOS Congress Lecture Hall 1
08:30-08:40 President's opening remarks
A. Diagnosis and treatment of borderline lesions
08:40-09:05 R. Fodde: Molecular background of desmoid tumours
09:05-10:15 Free Presentations
1 Desmoid tumors. A Clinical Review of 30 Patients with more than 20 years follow-up--Dalen
BPM, Goteborg, Sweden
2 Diagnostic impact of histologic parameters in differentiating enchondroma from grade I central
Chondrosarcoma--Hogendoorn PCW, Leiden, The Netherlands
3 BCL-2 Immunohistochemistry as a tool to distinguish osteochondroma and grade I peripheral
Chondrosarcoma--Bovee JVGM, Leiden, The Netherlands
4 The treatment of benign and low-grade malignant intramedullary chondroid tumors with
curettage and Cryosurgery--Schreuder HWB, Nijmegen, The Netherlands
5 The atypical Lipoma--Sommerville SMM, Birmingham, UK
10:15-10:45 Plenary selected poster review on borderline lesions: R. Veth
10:45-11:15 Coffee break and poster viewing
11:15-12:00 EMSOS lecture
J.C. Clevers: Fundamental mechanisms of tumourigenesis
12:00-12:15 Discussion
12:15-13:30 Lunch
B. Hereditary background in bone and soft-tissue tumours
13:30-13:55 M. Breuning: Overview of hereditary aspects of bone and soft-tissue tumours
13:55-15:15 Free presentations
22 Osteosarcoma subtypes in Osteosarcoma patients with multiple primary malignancies. Can rare
subtypes be indicative for cancer syndromes related with Osteosarcoma?--Hauben EI, Eindhoven,
The Netherlands
23 Gene and protein expression profiling in fibroblasts carrying a LI-Fraumeni p53 Germline muta-
tion that induces Chemoresistence to Doxorubicin--Sangiorgi L, Bologna, Italy
24 Somatic and Germical alteration of the TP53 Gene in pediatric Osteosarcoma--Patino A,
Pamplona, Spain
25 Chromosome 9 alterations and p16 expression in Central Chondrosarcomas--Rozeman L,
Leiden, The Netherlands
26 Identification and clinical validation of candidate target Genes from the amplified Region on
Chromosome Arm 17Q in malignant peripheral nerve sheath tumours--Smeland S, Oslo, Norway
27 Association between the occurance of Breast Cancer and cartilaginous tumors: Phenotopic
characterization of a Hitherto unrecognized potential hereditary trait--Cleton-Jansen AM, Leiden,
The Netherlands
15:15-15:45 Tea break and poster viewing
15:45-16:15 Plenary selected poster review on hereditary tumours: Stefan S. Bielack
S4 EMSOS Program
C. Surgery and soft tissue tumours
16.15-17.15 Free presentations
79 Reliability of risk factors and scoring systems in predicting fracturing in metastatic femoral bone
lesions--Linden YM van der, Leiden, The Netherlands
80 Predictors of wound complications following free and pedicled soft tissue flaps for reconstruction
after excision of soft tissue tumours--Abudu A, Birmingham, UK
81 Silver-coated tumor endoprostheses for prevention of deep infection--Gosheger G, Munster,
Germany
82 Our experience with the capanna method of Limb reconstruction using free fibular grafts and
cadaveric allograft report of 16 consecutive patients--Bickels J, Tel Aviv, Israel
17:15-17:45 Plenary selected poster review on Surgery and soft tissue tumours
Symposium of the EMSOS Nurse Group Lecture Hall 2
08:45-09:00 Opening: Cristiana Forni
G. Long-term side-effects after treatment in young adult survivors of childhood cancer
09:00-09:40 Guest speaker: N.E. Langeveld
127 Quality of life in young adults who are long-term survivors of childhood Bone Cancer
09:40-09:50 Discussion
Moderators: M.A.M. Kroeze, J. Ouwerkerk
09:50-10:10 128 The hidden Long-term effects of treatment--Riley V, London,UK
10:10-10.30 129 Functional outcome following Endo-prosthetic replacement (EPR) for Bone Tumours
involving the Knee--Williams C, Birmingham, UK
10:30-11:00 Coffee break and poster viewing
H. Nursing care for patients treated with chemotherapy for bone tumours
11:00-11:20 130 A structured and effective way to inform Sarcoma Patients--Leyerzapf NAC, Leiden, The
Netherlands
11:20-11:40 131 Nursing care for patients treated with Chemotherapy for Bone Tumours the "Euro-Ewing 99"
Protocol--Ouwerkerk ED, Leiden, The Netherlands
11:40-12:00 132 How to inform the New Sarcoma Patient?--Forni C, Bologna, Italy
12:00-12.20 133 Wound healing deficiencies after Major Tumour Operations--Lubben K, Munster, Germany
12:20-12:30 Discussion
12:30-14:00 Lunch
I. Psychosocial aspects for patients and family members
14:00-14:20 134 The role of Lymphedema treatment in the rehabilitation after Limb-sparing Surgery--Meller I,
Tel Aviv, Israel
14.20-14.40 135 The quality of interaction between the operators, the Oncological patient and his
family--Tanganelli F, Florence, Italy
14.40-15.00 136 Project for a school service--Forni C, Bologna, Italy
15.00-15.20 137 The experience of having Cancer--Woodford J, Middlesex, UK
15:20-15:45 Tea break and poster viewing
15:45-16:30 Guest speaker: N. Donders
138 The relation between Osteosarcoma, skiing and holidays for kids
16.30 Closing remarks
Friday, 24 May, 2002
EMSOS Congress
08:00-08:45 General assembly
08:45-09:00 Coffee break
D. Imaging of primary and recurrent tumours
09:00-09:25 J.L. Bloem: Imaging modalities of bone and soft-tissue tumours
EMSOS Program S5
09:25-10:30 Free communications
41 The role of functional nuclear scanning in the management of cartilaginous tumours--Choong
P, Melbourne, Australia
42 3-D imaging in planning and monitoring biological reconstructions in orthopaedic oncology: A
computer assisted approach. Future perspectives and preliminary results--Manfrini M, Bologna,
Italy
43 Value of dynamic contrast-enhanced MR imaging in diagnosing and classifying peripheral
vascular malformations--Rijswijk CSP van, Leiden, The Netherlands
44 Hip and Sacroileac joint infitration in sarcoma around the pelvis based on imaging and patho-
anatomical correlation studies and its implications on surgical strategy--Lindner NJ, Munster,
Germany
10:30-11:00 Coffee break and poster viewing
11:00-11:30 Plenary selected poster review on imaging: D. Vanel
11:30-11:50 Report of the Nurses' Symposium
E. Chemotherapy and other
11:50-12:35 Free presentations
98 Comparison of Doxorubicine versus VP16-ifosfamide in addition of high dose methotrexate as
pre-operative Chemotherapy in Osteosarcomas. A randomized trial by the French Society of
Pediatric Oncology--Brugieres LB, Villejuif, France
99 Gene therapy of Osteosarcoma: Targeting Adenoviruses towards integrins increases transduction
efficiency and tumor cell kill in vitro and in vivo--Witlox MA, Amsterdam, The Netherlands
100 Ewing tumor (ET) of the Spine - experience of the cooperative Ewing's sarcoma studies
(EI)CESS 81-92--Ahrens S, Munster, Germany
12:35-13:45 Lunch
13:45-14:15 Plenary selected poster review on Chemo and other: P. Picci
14:15-15:00 Campanacci lecture
O. Steen Nielsen: Progress in the treatment of bone and soft-tissue tumours of the extremities
15:00-15:15 Discussion
15:15-15:45 Tea break and poster viewing
F. Upper extremity reconstructions
15.45-16.45 Free presentations
54 Resection in Primary-malignant scapula tumors--Taminiau AHM, Leiden, The Netherlands
55 Upper Limb reconstruction in Bone Sarcomas of childhood--Manfrini M, Bologna, Italy
56 Upper extremity reconstruction: Is Limb-salvage for Osteosarcoma of the proximal humerus a
"Safe" procedure?--Bielack S, Munster, Germany
57 Cemented modular prosthesis in the proximal humerus: long term follow-up--Donati M,
Bologna, Italy
16:45-17:15 Plenary selected poster review on upper extremity reconstructions: R. Grimer
17.15-17.45 Discussion
17.45-17.55 Closing remarks

				

